# Left Ventricular Thrombus Despite Prescribed Direct Oral Anticoagulant Therapy in Chronic Heart Failure With Reduced Ejection Fraction: A Case Report and Review of Risk Stratification

**DOI:** 10.7759/cureus.113208

**Published:** 2026-07-23

**Authors:** Gemechu Ayana, Lucas Garcia Reinoso, Baziliya Keraga, Isabel Conde, Goitom Weldearegay, Leah Ragbir, Wazema Desta, Minase Temesgen, Melat Demisse, Cristina A Mitre

**Affiliations:** 1 Internal Medicine, SUNY Downstate University Hospital, New York City, USA; 2 Internal Medicine, SUNY Downstate Health Sciences University, New York City, USA; 3 Internal Medicine, Jimma University, Jimma, ETH; 4 Cardiology, Veterans Affairs New York Harbor Healthcare System, Brooklyn Campus, Brooklyn, USA

**Keywords:** anticoagulation, apical aneurysm, cardiac magnetic resonance imaging (cmr), direct oral anticoagulants (doacs), heart failure with reduced ejection fraction (hfref), left ventricular thrombus, precision medicine, risk stratification

## Abstract

Left ventricular thrombus (LVT) remains a clinically important complication associated with increased risks of systemic thromboembolism, ischemic stroke, and adverse cardiovascular outcomes. The optimal duration of anticoagulation treatment remains difficult to establish, and it should be individualized depending on patient characteristics.

We present the case of a 65-year-old man with a history of coronary artery disease (CAD) status post-myocardial infarction, who underwent coronary bypass graft surgery, ischemic cardiomyopathy, chronic heart failure with reduced ejection fraction (HFrEF) with left ventricular ejection fraction of 30%-35% diagnosed in 2022, atrial flutter status post-ablation, and chronic kidney disease stage II who was found to have LV apical aneurysm with a newly developed left ventricular apical thrombus on transthoracic echocardiogram (TTE). The patient had been prescribed apixaban for stroke prevention; however, he reported inconsistent medication adherence. A repeat TTE a few months later revealed the apical thrombus again despite prescribed anticoagulation. The patient remained asymptomatic. Cardiac magnetic resonance imaging (CMR) was planned for further thrombus characterization and risk assessment.

This case highlights the occurrence of LVT in chronic HFrEF outside the traditional post-myocardial infarction setting and emphasizes the importance of medication adherence in preventing thrombus formation. We review current evidence regarding LVT management, emerging data supporting direct oral anticoagulants (DOACs), and factors associated with thrombus persistence and recurrence. Additionally, we discuss evolving approaches to risk stratification incorporating thrombus morphology, ventricular remodeling, artificial intelligence (AI)-assisted imaging, and genomic risk assessment. This case underscores the limitations of generalized treatment recommendations and highlights the need for individualized, risk-adapted management strategies to optimize anticoagulation duration and reduce thromboembolic complications in patients with LVT.

## Introduction

Left ventricular thrombus (LVT) remains a clinically important complication associated with substantial thromboembolic morbidity despite advances in reperfusion therapy and contemporary heart failure management [1-5]. LVT typically develops in the setting of left ventricular systolic dysfunction, regional wall-motion abnormalities, and intracavitary blood stasis, most commonly following large anterior ST-segment elevation myocardial infarction (STEMI), although increasing recognition in nonischemic cardiomyopathies has broadened its clinical significance [1,4,6-9].

Current American College of Cardiology and American Heart Association recommendations support anticoagulation for approximately three months with repeat imaging to confirm thrombus resolution [8-11]. However, important uncertainties remain regarding the optimal duration of therapy, particularly in patients with persistent ventricular dysfunction, recurrent thrombus formation, or thrombus development unrelated to acute myocardial infarction [8-18]. Existing literature demonstrates that recurrence of LVT and thromboembolic events may occur even after apparent thrombus resolution and discontinuation of anticoagulation [19]. Some studies suggest that embolic risk persists beyond six months, raising questions regarding whether selected patients may benefit from prolonged or individualized anticoagulation strategies [20]. Although contemporary studies support the use of direct oral anticoagulants (DOACs) as alternatives to vitamin K antagonists (VKAs) [12-20], current treatment decisions largely remain based on generalized recommendations rather than patient-specific risk assessment. Emerging evidence suggests that thrombus morphology, imaging characteristics, clinical variables, artificial intelligence (AI)-assisted image analysis, and genomic risk factors may identify patient subgroups at increased risk for persistence, recurrence, and thromboembolic complications [17].

Although contemporary guidelines recommend anticoagulation followed by interval imaging, important uncertainties remain regarding management of patients who develop LVT despite prescribed anticoagulation, particularly in the setting of chronic left ventricular dysfunction unrelated to acute myocardial infarction. Medication adherence, thrombus morphology, and persistent ventricular remodeling may substantially influence recurrence risk and optimal treatment duration.

We present a case of incidentally identified LVT in a patient with ischemic cardiomyopathy, chronic heart failure with reduced ejection fraction (HFrEF) diagnosed in 2022, atrial flutter status post flutter line ablation, and stage II chronic kidney disease who developed an apical thrombus still present after a few months of anticoagulation, with inconsistent medication adherence identified as a likely contributing factor. This case highlights evolving patterns of LVT outside the traditional post-ST-segment elevation myocardial infarction (STEMI) setting and underscores the need for individualized, risk-adapted management strategies that integrate clinical, imaging, and emerging precision medicine approaches.

## Case presentation

The patient is a 65-year-old man with a past medical history significant for ischemic cardiomyopathy with LV apical aneurysm, chronic heart failure with reduced ejection fraction (HFrEF) with left ventricular ejection fraction (LVEF) of 30%-35% diagnosed in 2022, atrial flutter status post flutter line, and stage II chronic kidney disease (baseline creatinine: 1.7 mg/dL). He started treatment for heart failure, and he had improvement in LV systolic function from an initial LVEF of 30%-35% to 40%-45% on transthoracic echocardiogram (TTE) done in October 2024, although an apical aneurysm remained present, with no LV apical thrombus reported.

The patient had regular follow-up in the outpatient cardiology clinic at the VA NY Harbor Healthcare System-Brooklyn for his chronic cardiovascular conditions. His medications included carvedilol 6.25 mg twice daily, sacubitril/valsartan 49/51 mg twice daily, empagliflozin 10 mg daily, atorvastatin 40 mg daily, apixaban 5 mg twice a day, and aspirin 81 mg daily.

He denied chest pain, dyspnea, orthopnea, paroxysmal nocturnal dyspnea, lower extremity edema, palpitations, syncope, focal neurologic deficits, constitutional symptoms, or recent hospitalizations. Functionally, he was classified as New York Heart Association (NYHA) class I. Review of systems was otherwise negative, and physical examination was unremarkable.

**Figure 1:**
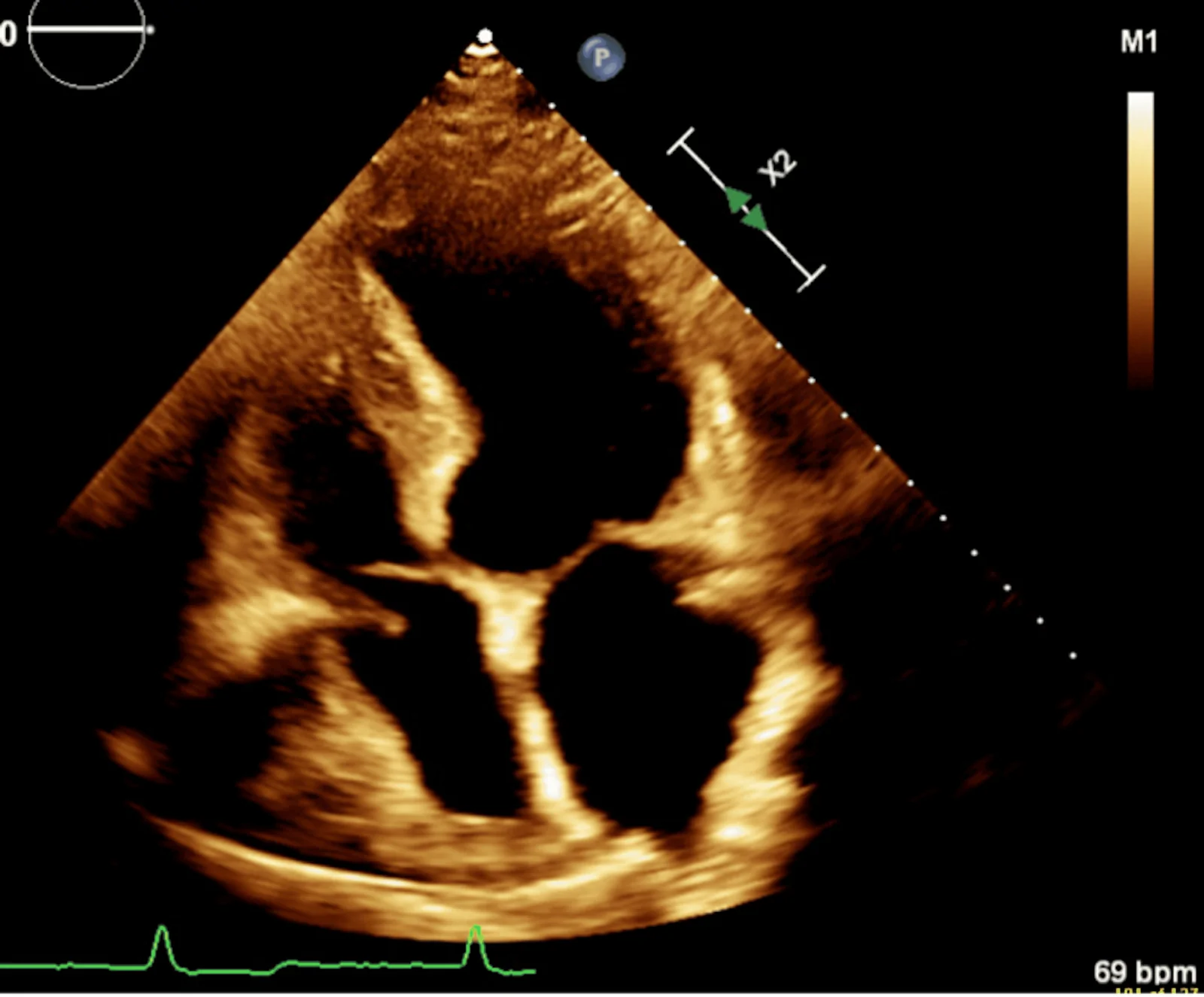
TTE in October 2025 (without ultrasound contrast agent) (A4C view) LV apex not well-visualized, suspected apical thrombus TTE: transthoracic echocardiography, A4C: apical four-chamber, LV: left ventricle

**Figure 2:**
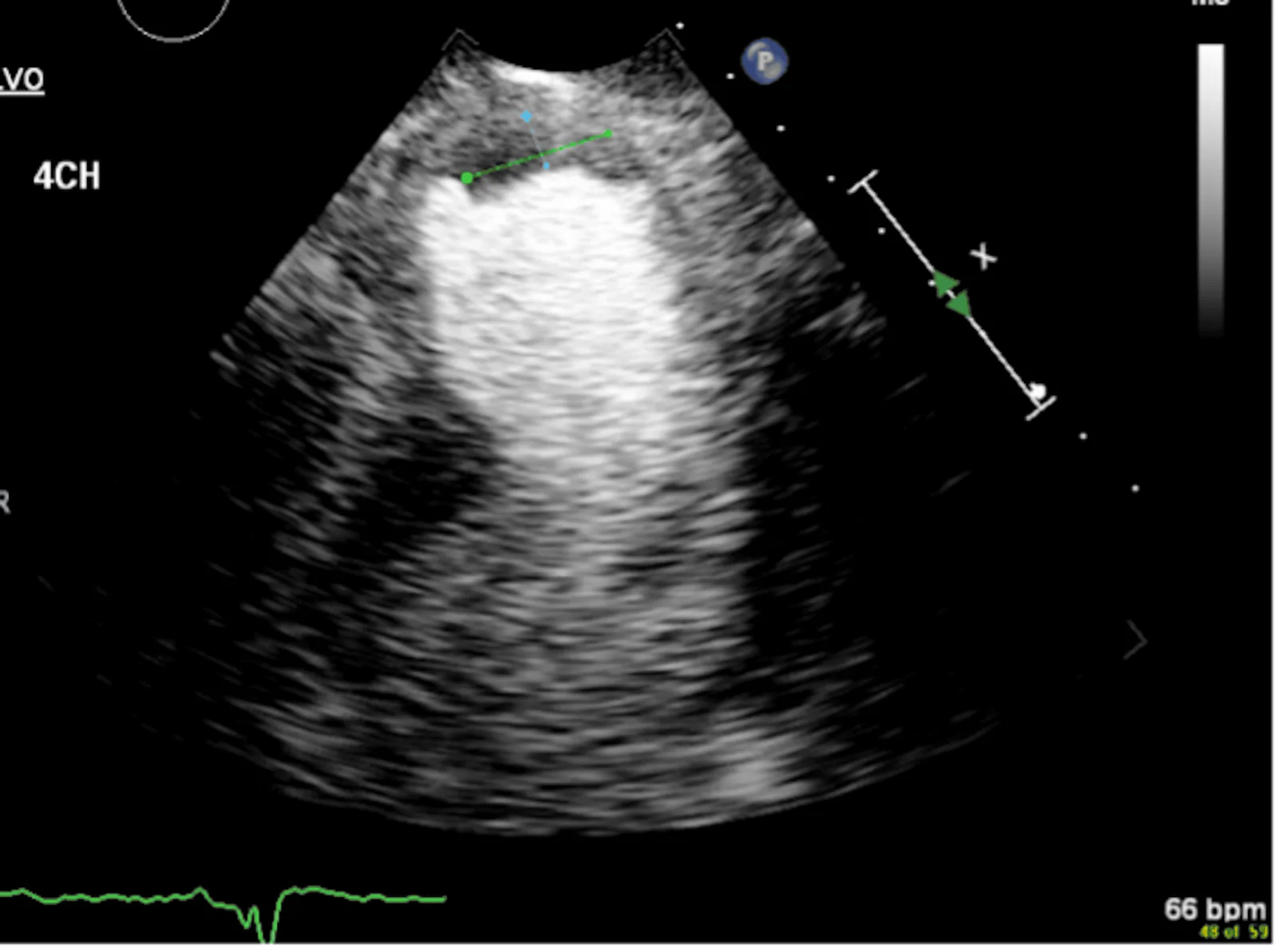
TTE in October 2025 with contrast (Definity) (A4C view) Left ventricle: mild to moderately decreased systolic function. Ejection fraction is mild to moderately decreased. The estimated ejection fraction is 40%-45%. Apex and apical segments are akinetic. Mild basal infero-septal severely hypokinetic. With contrast injection, an echolucency approximately 0.7 x 2 cm is visualized in the LV apex. TTE: transthoracic echocardiography, A4C: apical four-chamber, LV: left ventricle

A TTE performed one year later, in October 2025, reported borderline decreased systolic function with an aneurysmal LV apex that was not well visualized (Figure 1), which led to a recommendation for a repeat limited echocardiogram using ultrasound contrast agent Definity (perflutren lipid microsphere). A contrast-enhanced transthoracic echocardiogram (TTE) using perflutren lipid microsphere (Definity) performed days later demonstrated mildly to moderately reduced left ventricular systolic function with an estimated LVEF of 40%-45% and confirmed an apical left ventricular thrombus with improved delineation of the left ventricular endocardial borders (Figure 2). The patient was subsequently started on therapeutic anticoagulation with apixaban.

**Figure 3:**
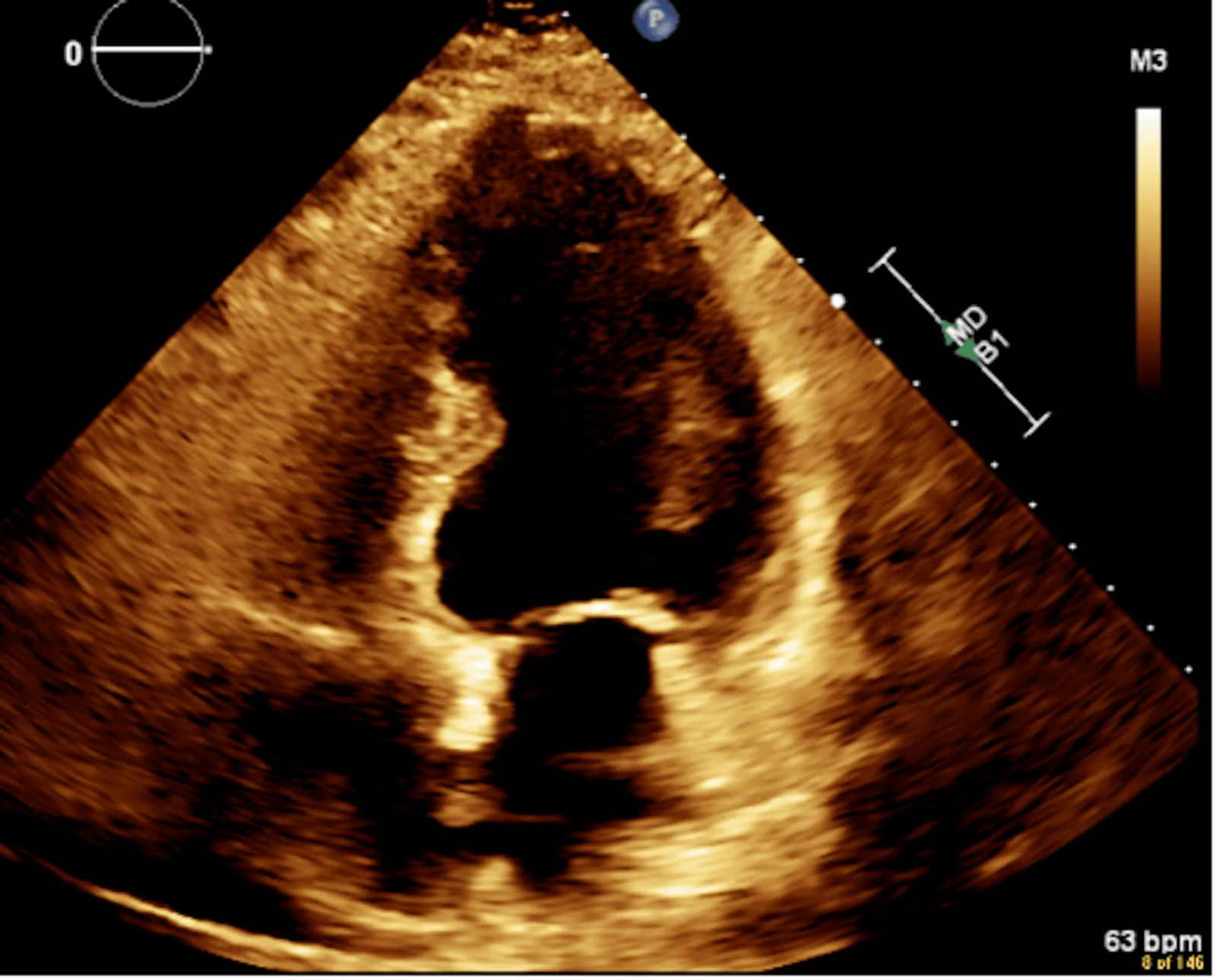
Repeat TTE in April 2026 without ultrasound contrast agent (A4C view) Apical thrombus still present TTE: transthoracic echocardiography, A4C: apical four-chamber, LV: left ventricle

**Figure 4:**
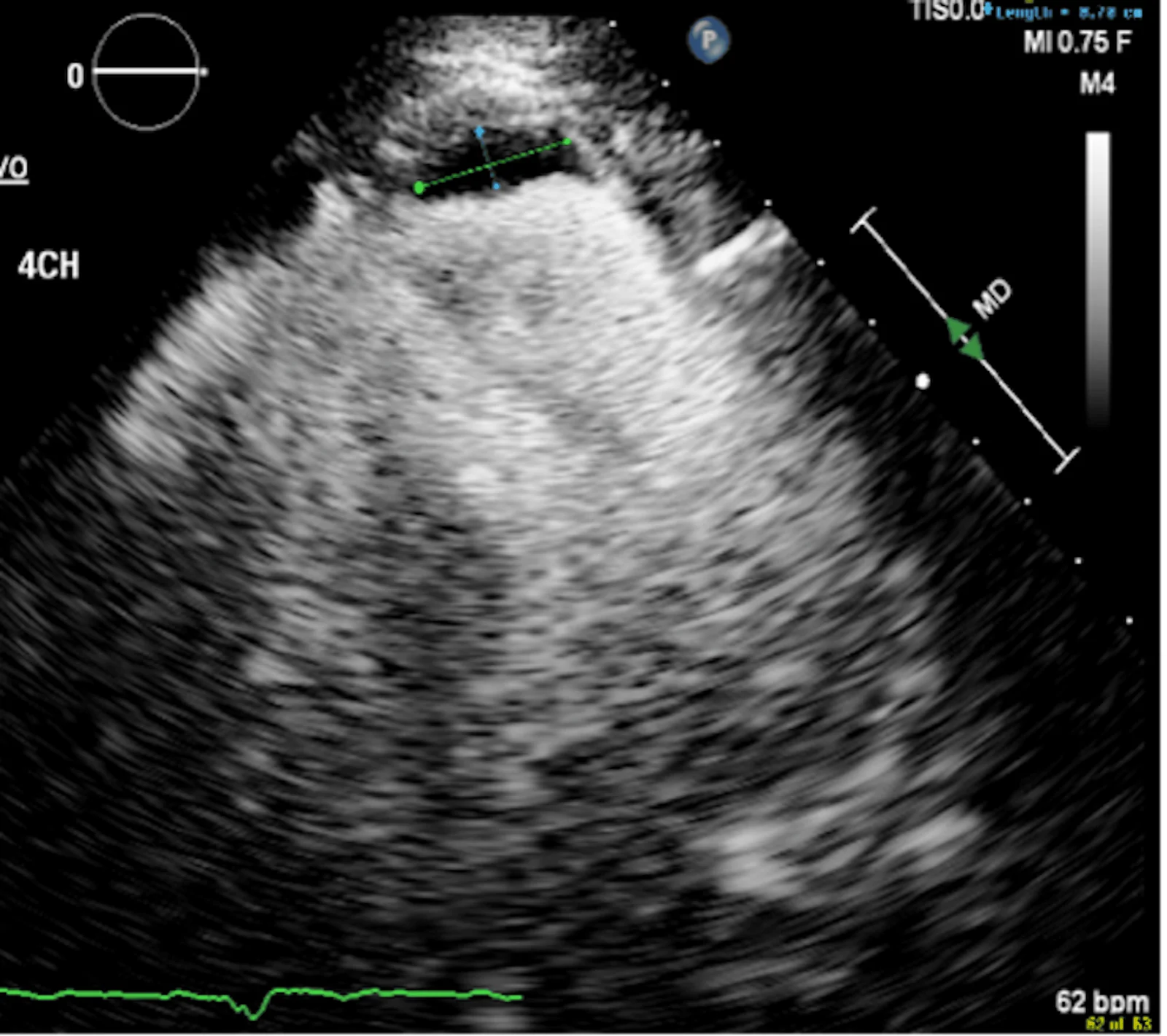
Repeat TTE in April 2026 with contrast agent (Definity) (A4C view) Apical thrombus still present TTE: transthoracic echocardiogram, A4C: apical four-chamber, LV: left ventricle

A follow-up TTE approximately six months later, performed in April 2026 (Figure 3 and Figure 4), demonstrated persistent mildly to moderately reduced left ventricular systolic function with an estimated LVEF of 40%-45% and a persistent left ventricular apical thrombus despite anticoagulation.

The patient remained asymptomatic despite persistent thrombus on full-dose apixaban, although he acknowledged inconsistent medication adherence during the initial three months after diagnosis of LV apical thrombus. Cardiac magnetic resonance imaging (CMR) was planned to further characterize thrombus morphology, evaluate the apical aneurysm, and guide subsequent management decisions. Cardiothoracic surgery consultation was obtained to assess potential therapeutic options should high-risk features be identified.

The development of LVT in this patient raised several clinically relevant questions. Although suboptimal adherence to apixaban likely contributed to thrombus persistence, this case also highlights broader uncertainties frequently encountered in clinical practice regarding the identification of patients at increased risk for thrombus persistence, recurrence, and future thromboembolic complications.

Unlike the classic presentation of LVT following an acute anterior ST-segment elevation myocardial infarction (STEMI), this patient developed thrombus in the setting of chronic HFrEF with persistent systolic dysfunction, with thrombus remaining present after six months of prescribed therapeutic anticoagulation. The case illustrates the limitations of current generalized treatment recommendations and emphasizes the importance of incorporating patient-specific clinical characteristics, thrombus morphology, ventricular remodeling, adherence to prescribed medical therapy, and anticoagulation-related factors into decisions regarding the optimal duration of anticoagulation and follow-up imaging.

## Discussion

Current management of left ventricular thrombus

LVT management focuses on reducing thromboembolic complications while balancing bleeding risk. Prompt identification and initiation of anticoagulation remain the cornerstone of therapy because untreated LVT is associated with substantial risks of ischemic stroke, systemic embolization, and adverse cardiovascular outcomes [3,6-8].

Accurate diagnosis is central to management. Transthoracic echocardiography (TTE) remains the first-line imaging modality because of its broad availability and bedside accessibility; however, sensitivity may be limited in patients with apical abnormalities or poor acoustic windows [2,3,10]. Use of contrast-enhanced echocardiography improves endocardial border definition and increases diagnostic yield [10,11]. Cardiac magnetic resonance imaging (CMR) with late gadolinium enhancement remains the reference standard because of superior sensitivity, specificity, and ability to characterize thrombus morphology and myocardial viability [2-4].

Historically, VKAs such as warfarin represented standard therapy due to evidence demonstrating reductions in embolic events and thrombus persistence [7]. Current American Heart Association and American College of Cardiology recommendations generally support initiating therapeutic anticoagulation for approximately three months following LVT diagnosis with repeat imaging to document thrombus resolution [8,11]. For patients with persistent thrombus, severe left ventricular dysfunction, ventricular aneurysm, or recurrent thrombotic risk factors, extended treatment durations of three to six months or longer may be considered through individualized decision-making [8].

Despite these recommendations, optimal anticoagulation duration remains uncertain. Existing guidance largely derives from observational studies and expert consensus rather than prospective randomized trials [8]. Patients with remote myocardial infarction, chronic ischemic cardiomyopathy, or persistent ventricular dysfunction present management challenges because thromboembolic risk may continue beyond apparent thrombus resolution [19,21].

DOACs have increasingly emerged as alternatives to warfarin because of predictable pharmacokinetics, fewer drug and dietary interactions, and lack of routine laboratory monitoring. Multiple observational studies, systematic reviews, and contemporary meta-analyses demonstrate comparable efficacy and safety between DOACs and VKAs for thrombus resolution and prevention of systemic embolization [9,12-20]. More recent prospective and randomized studies further support the non-inferiority of DOAC therapy [14,15,20,22-24]. Ongoing trials, including RELEVENT, may provide additional evidence regarding optimal anticoagulant selection and future treatment approaches [23].

Risk stratification of patients with left ventricular thrombus

Current management of LVT largely relies on thrombus resolution and improvement in ventricular function to guide anticoagulation duration. However, available evidence suggests substantial heterogeneity exists among patients with LVT regarding thrombus persistence, recurrence, and risk of future thromboembolic events [8,19]. This variability suggests that risk stratification models may help identify patient subgroups who could benefit from individualized anticoagulation duration and surveillance strategies.

Recurrence of LVT carries potentially devastating consequences because recurrent thrombus formation has been associated with significantly increased rates of cardioembolic events, particularly ischemic stroke [19]. In patients with post-myocardial infarction LVT, thrombus protrusion, recurrent thrombus formation, and failure of initial thrombus resolution have been identified as independent predictors of subsequent ischemic stroke [16]. Namjouyan et al. additionally demonstrated that despite initial thrombus resolution, approximately 5.2% of patients subsequently experienced acute ischemic stroke, with cardioembolic mechanisms accounting for most events [19]. Multivessel coronary artery disease was similarly associated with greater recurrence risk compared with single-vessel disease [19].

Beyond clinical variables, echocardiographic findings may provide important prognostic information. Studies have demonstrated associations between persistent LVT and larger left ventricular end-diastolic diameter (LVEDD), larger left ventricular end-systolic diameter (LVESD), increased left atrial volume, reduced left ventricular wall-motion scores, and higher rates of apical aneurysm formation [16]. These findings suggest that advanced ventricular remodeling and impaired ventricular mechanics may contribute to thrombus persistence.

Morphologic characteristics of the thrombus may further refine risk assessment. Thrombus size, shape, mobility, and protrusion have all demonstrated associations with both embolic risk and likelihood of thrombus resolution [8,16]. Smaller baseline thrombus size appears associated with an increased probability of thrombus regression, whereas larger thrombus burden may correlate with greater ventricular dysfunction and chamber enlargement. Some studies suggest that each 1 mm increase in thrombus size may be associated with slower rates of thrombus resolution [16].

Among imaging characteristics, thrombus mobility appears particularly important. Oh et al. demonstrated that mobility represented the strongest independent predictor of early thrombus resolution [16]. Mobile protuberant thrombi may provide a greater surface area for interaction with endogenous fibrinolytic pathways and anticoagulant agents. Round thrombi projecting into the ventricular cavity similarly demonstrated earlier resolution compared with mural thrombi, potentially reflecting differences in thrombus mobility and exposed surface area [16].

Collectively, integration of clinical characteristics, ventricular remodeling parameters, and thrombus morphology may provide the foundation for future predictive models capable of identifying low- and high-risk LVT subgroups. Such risk stratification tools may ultimately facilitate individualized anticoagulation duration and surveillance strategies beyond current generalized recommendations.

Artificial intelligence, genomics, and future directions

Current LVT management relies primarily on thrombus resolution and recovery of ventricular function to guide anticoagulation duration. However, clinical characteristics, thrombus morphology, and advanced imaging findings may help identify patients at increased risk for persistence, recurrence, and thromboembolic events. Emerging artificial intelligence (AI) applications may improve standardization of image acquisition and analysis, enabling more consistent assessment of thrombus burden, ventricular remodeling, and high-risk imaging features [25,26]. In parallel, genomic technologies such as next-generation sequencing (NGS) and polygenic risk scores (PRS) have shown promise in cardiovascular risk prediction and may eventually contribute to individualized thrombotic risk assessment [6,27-31]. Although evidence supporting routine use of AI, genetic testing, or pharmacogenomics in LVT remains limited, integration of imaging, clinical, and genomic data may offer future opportunities for personalized anticoagulation strategies and improved risk stratification [8,22,30-32].

Use of pharmacogenetics in DOAC prescriptions

DOACs have a broad therapeutic index and generally demonstrate improved safety profiles compared with VKAs; however, clinically significant bleeding and subtherapeutic anticoagulation remain important concerns despite their increasing use in LVT management [12-15,17,18,20,24]. As anticoagulation strategies continue to evolve, precision medicine approaches such as pharmacogenomics may eventually provide additional tools for individualized risk reduction. Interindividual variability in drug metabolism and response remains incompletely understood and may contribute to differences in anticoagulant efficacy and adverse events. In addition to conventional clinical factors, genetic variants, epigenetic mechanisms, and environmental influences may contribute to variability in therapeutic response. Future investigations integrating genomic data with clinical characteristics may improve individualized anticoagulation selection and treatment strategies in patients with LVT [8,22].

Table 1 presents proposed high-risk features associated with left ventricular thrombus and their potential clinical significance.

**Table 1:** Proposed high-risk features associated with LVT and their potential clinical implications Our patient demonstrated multiple proposed high-risk features, including reduced left ventricular systolic function and anticoagulation-related risk factors, supporting the concept that future risk stratification models may improve individualized LVT management. LV: left ventricular, LVEF: left ventricular ejection fraction, LVT: left ventricular thrombus

Category	High-risk feature	Potential clinical significance	References
Thrombus morphology	Mobile or protruding thrombus	Increased embolic potential and higher risk of systemic thromboembolism	[5,7,16]
Thrombus characteristics	Large thrombus burden	Greater likelihood of thrombus persistence and embolic complications	[1,7,8]
Thrombus characteristics	Persistent LVT despite anticoagulation	Associated with delayed resolution and recurrent adverse events	[19,21]
Thrombus characteristics	Recurrent LVT apparent resolution	Increased risk of future thromboembolic events	[19,21]
Ventricular function	Severely reduced LVEF (<30%-35%)	Promotes blood stasis and thrombus formation	[1,4,8]
Ventricular remodeling	Apical akinesis/dyskinesia or LV aneurysm	Creates regions of stagnant flow favoring thrombus development	[1,4,6]
Clinical history	Prior stroke or systemic embolic events	May indicate increased future embolic risk	[5,7]
Anticoagulation factors	Premature cessation or inadequate anticoagulation	Increased risk of thrombus persistence or recurrence	[19,21]
Imaging findings	Delayed thrombus resolution on serial imaging	May identify patients requiring prolonged therapy	[8,16]
Emerging risk markers	Artificial intelligence-derived imaging features	Potential future enhancement of imaging standardization and risk prediction	[25,26]
Emerging risk markers	Genomic or polygenic risk profiles	Potential individualized prediction of recurrence and treatment response	[6,8,27-33]

This case report has several limitations. As a retrospective case report of a single patient, its findings are not generalizable. In addition, objective measures of anticoagulant adherence and comprehensive serial imaging data were unavailable, limiting definitive conclusions regarding direct oral anticoagulant treatment failure. Finally, the discussion of artificial intelligence, genomics, and pharmacogenetics is intended to highlight emerging areas of investigation rather than approaches validated by this case.

## Conclusions

Left ventricular thrombus remains a clinically important complication associated with significant thromboembolic risk. This case demonstrates that LVT may persist despite prescribed DOAC therapy in the setting of suboptimal medication adherence, highlighting the importance of adherence and serial imaging. Further studies are needed to define optimal anticoagulation strategies and improve risk stratification for patients with persistent or recurrent LVT.
